# Challenges in Removing the Essure^®^ Device

**DOI:** 10.1155/2020/8823606

**Published:** 2020-08-24

**Authors:** Daniëlle M. van Gastel, Liselotte W. Maassen, Carolien A. M. Koks, Sebastiaan Veersema, Marlies Y. Bongers

**Affiliations:** ^1^Máxima Medical Center Veldhoven, De Run 4600, 5504 DB Veldhoven, Netherlands; ^2^Meander Medisch Centrum, Amersfoort Maatweg 3, 3813 TZ Amersfoort, Netherlands; ^3^University Medical Center Utrecht, Heidelberglaan 100, 3584 CX Utrecht, Netherlands; ^4^University Maastricht, Researchschool Grow, P. Debyelaan 25, 6229 HX Maastricht, Netherlands

## Abstract

We present a case about Essure^®^ removal surgery in which the third markers of the device have torn off. The woman needed a second surgery for complete removal of the devices. Fluoroscopy during surgery is a good method to visualize the lost fragments. With fluoroscopy, a hysterectomy is not needed for complete removal. It is important to understand the structure of the device and to be aware of the four radiopaque markers during surgery and their removal.

## 1. Introduction

**Figure 1:**
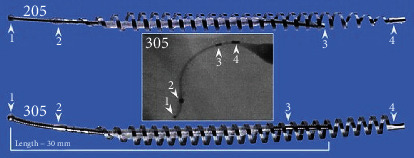
Essure^®^ (Bayer AG, Leverkusen, Germany) with the four radiopaque markers (1). 1: distal end of the inner coil, 2: distal end of outer coil, 3: proximal end of the inner coil, and 4: proximal length of the outer coil [4].

**Figure 2:**
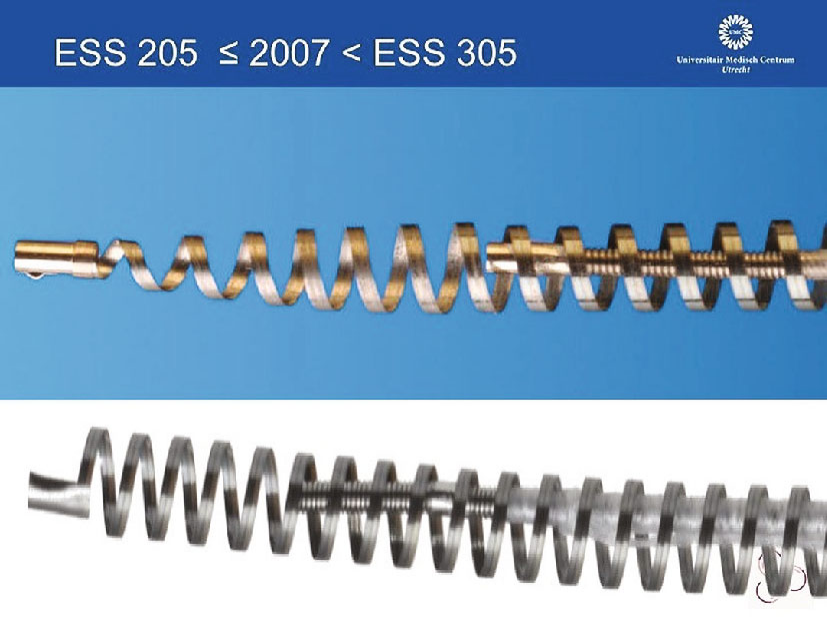
Difference of the third maker between ESS 205 and ESS 305.

In November 2002, the United States Food and Drug Administration (FDA) approved Essure® (Bayer AG, Leverkusen, Germany) as the first hysteroscopic tubal occlusion device for permanent female sterilization. [1] Essure^®^ is an expanding device that is placed into the fallopian tube. This microinsert consists of two coils. The inner coil is made from stainless steel and polyethylene terephthalate (PET) fibers. The outer coil consists of a nickel-titanium alloy (nitinol). [1, 2]. There are four radiopaque markers present on the device; the first marker is at the distal end of the inner coil. The second marker is the place where the outer coil starts and is connected with the inner coil. The third marker is at the proximal end of the inner coil, and the fourth one is at the proximal end of the outer coil (Figures 1 and 2). [2, 3] The earlier model, ESS205, was well tolerated by patients. However, the device had some disadvantages, and therefore, a new device, ESS305, was launched in 2007. For the production of this new device, the same materials were used. [4].

In the last years, increasing problems with the Essure® sterilization devices have been reported. [5] Women report multiple symptoms as abdominal or pelvic pain, pain in hips or groins, fatigue, abnormal menstrual bleeding, and skin problems. Until now, increasing number of women contact their gynecologist with the request for surgical removal. In 2006, the first article concerning Essure^®^ removal techniques was published. Hereafter, more literature appeared. Albright et al. recommended in 2013 to avoid cauterization and stretching of the microinsert to prevent tearing. They also state that it is important to assess complete removal of the inserts at the end of the procedure. [6] Bayer recently reviewed the instructions for use (IFU) of the Essure® ESS305. Information regarding appropriate removal of the devices was added. They advise to start the procedure with hysteroscopy and to remove the most proximal part of the outer coil (the fourth marker), if visible. [7] This may facilitate laparoscopic removal of the device, as the fourth marker is the biggest part of the Essure^®^. Also, hysteroscopic removal of the fourth marker prevents the device from tearing.

We present a case of Essure^®^ ESS205 removal where the third radiopaque marker teared off. Until now, it was unknown that not only the fourth marker but also the third marker of the device was prone to fragmentation. A second laparoscopy with intraoperative fluoroscopy was used to localize and remove the retained part of the devices successfully. The goal of this case report is to create awareness of the differences between the ESS205 and ESS305 models in surgical removal.

## 2. Case Presentation

A 46-year-old woman visited the outpatient clinic in May 2016 of a large teaching hospital in The Netherlands for evaluation of symptoms following Essure^®^ sterilization. Her main symptoms were headache, abdominal pain, lower back pain, and pelvic pain. Her medical history was notable for hypertrophic cardiomyopathy, an implantable cardioverter-defibrillator (ICD) and shoulder and lower back myalgia. She used the following medication: acenocoumarin, furosemide, esomeprazole, nitroling, salbutamol, and verapamil. She had known allergies for nickel, antimycotics, protone pump inhibitors, amoxicillin, macrolides, and tetracyclines. Essure^®^ sterilization was successfully performed in 2007, using the ESS205 model. The procedure went uncomplicated in an outpatient setting, and the patient went home on the same day. After three months, tubal occlusion was confirmed by hysterosalpingogram (HSG).

**Figure 3:**
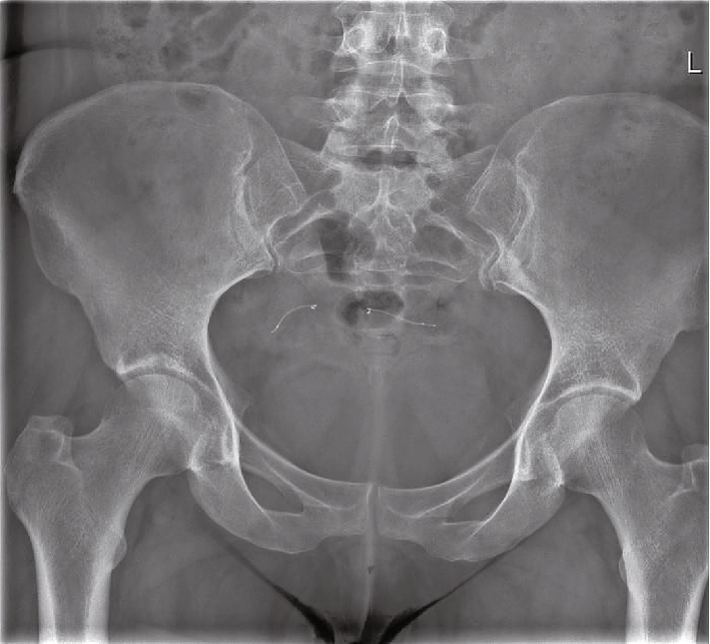
Flat panel abdominal X-ray (anterior posterior view) showing both implants (ESS 205) with symmetrical deployment in the pelvic area and the proximal markers are 25 mm apart.

During gynecologic examination in May 2016, no abnormalities were found. Both devices seemed to be in the right position, assessed by transvaginal ultrasound imaging. She obtained a flat panel abdominal X-ray showing both implants with symmetrical deployment in the pelvic area and the proximal markers 25 mm apart (Figure 3). The woman requested surgical removal of the devices. Because of her complex medical history and high risk for complications, the team hesitated to operate. However, she persisted in her wish and was scheduled for removal of the devices.

Six weeks later, the patient underwent a hysteroscopy followed by laparoscopy. During surgery, her ICD was turned off. At hysteroscopy, a normal uterine cavity was seen and both tubal ostia were visualized. There were no visible parts of the devices in the uterine cavity; both ostia where dilated with a grasper. During laparoscopy, no adhesions, endometriosis, or any other pelvic or abdominal pathology were seen. Both microinserts were visible in the fallopian tubes; there were no signs of perforation or incorrect positioning of the implants. At first, a linear incision over the corneal segment of the right tube was made with needle monopolar electrosurgery, followed by gently pulling the proximal end of the Essure^®^ with a grasper. The inner and outer coils were stretched out but easily and completely removed, including the fourth marker. Hereafter, salpingectomy was performed. On the contralateral side, the same technique was performed. The inner coil was stretched out but removed easily; the outer coil was also stretched out but broke into two segments. The remainder was left in the fallopian tube while continuing with the salpingectomy. The other segment was removed in parts from the intrauterine part of the tube but after thorough searching, the fourth marker could not be found. Hysteroscopy was repeated. While opening the left tubal ostia with a grasper, the fourth marker was seen and removed. Hemostasis was adequate. The removed microinserts were closely inspected after laparoscopy to verify the presence of the most distal end of the outer coil. Both inserts seemed to be complete. No complications occurred, and the patient went home the same day. Her postoperative recovery was uncomplicated.

**Figure 4:**
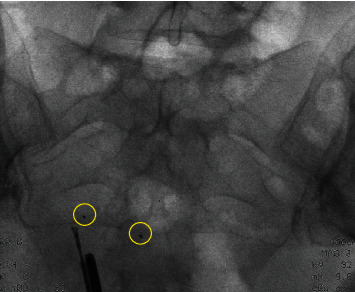
Fluoroscopy during surgery. Two markers are visible.

**Figure 5:**
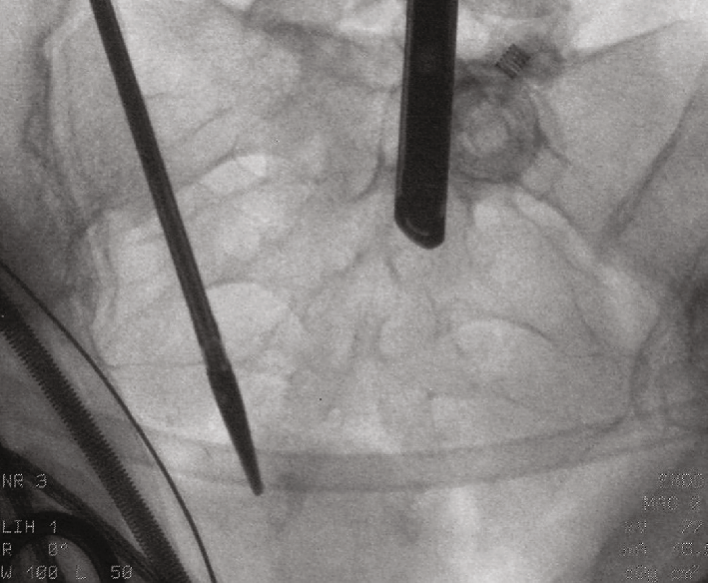
Fluoroscopy during surgery. The markers are removed.

Five weeks later, she returned for her postoperative control visit. Her abdominal pain was reduced, but still present. Also, she still experienced fatigue and was not satisfied. The doctor suggested that her complaints were probably not caused by Essure^®^. Due to persistent symptoms, she returned 17 months after the initial removal surgery. A flat panel abdominal X-ray was performed and showed two metal fragments. These were most likely to be parts of the removed Essure^®^ devices. During initial laparoscopy, both fourth markers were visualized and removed, implying that these fragments were different parts of the device, supposedly the third markers (proximal marker of inner coil). The woman requested removal of these fragments. Six weeks later, hysteroscopy and laparoscopy were repeated. Hysteroscopically, no fragments of the devices were seen. During laparoscopy, intraoperative fluoroscopy was performed to visualize both remnants. The markers were bilaterally removed from the cornua, and this resulted in complete removal of the device remnants (Figures 4 and 5). The postoperative period was uncomplicated. Six week later, the woman continued to do well. Her symptoms were reduced; she only continued to experience symptoms of her left groin.

## 3. Discussion

Few case-reports about the removal of the fourth marker are published. [8, 9] This is, by our knowledge, the first report written about fragmentation of the third marker. Until now, it was not recognized that the third marker could also be teared off during removal surgery.

Prior to removal surgery, it is important to understand the structure of the device. The inner coil consists of stainless steel and polyethylene terephthalate (PET) fibers running along the inner coil. The outer coil consists of nickel-titanium alloy (nitinol). [1, 10] The ESS205 and ESS305 are made from the same materials. Concerning the third marker, there is a difference between the two devices. The third marker of ESS205 is located at the proximal end of the inner coil. The third marker of ESS305 is situated a few millimeters more distal from the end. The third marker of ESS205 is prone to fragmentation after stretching the device during removal surgery, because of its position on the device, where this is less likely to happen with ESS305 because the marker is not terminal. The difference between the proximal part of the outer coil is that there is a little tube at the end of ESS205 that is placed over the nitinol coil. At ESS305 is a platinum plate attached at the end of the outer coil. In both models, the fourth marker is the biggest part of the device and prone to fragmentation. The PET fibers of the inner coil of Essure^®^ react with the fallopian tube tissue which induces fibrosis. The outer coil becomes frail after some time, and traction may cause it to break. The most proximal part of the outer coil with the fourth marker is easily lost and can be hard to find. [6] In summary, not only the fourth marker but also the third marker of the ESS205 can tear off and can easily be lost during removal surgery.

Intraoperative fluoroscopy is described in previous case reports to find lost fragments of the insert. [8, 11] It is a challenge to localize the exact localization of the fragment. Even with fluoroscopy, technical skills are needed for the removal. In view of the small size of the fragments and the limits of the human eye, this and other cases elaborate that there should be a low threshold for the use of intraoperative fluoroscopy. This technique can prevent the need for reoperation and eventually hysterectomy.

Increasing concerns regarding potential adverse events of Essure^®^ has led many women to seek removal of the device. In the absence of an Essure^®^ patient registry and the frequency of adverse events, there is no knowledge of an absolute number of women suffering from these symptoms. Literature published on the microinsert consists of nonrandomized clinical trials and case reports. There appears to be no clear consensus of the best way to remove Essure^®^ devices in women who subsequently develop symptoms possibly related to the device. In the first 12 weeks after placement, hysteroscopic removal is possible. [6] The risks associated with this method include tearing of the device within the tube, as well as inducing tubal trauma, which may not be noticed during hysteroscopy. Thorough inspection of the removed device is important. For the majority of women, hysteroscopic removal is not possible, since a lot of the devices have been in situ for more than years and have grown into the tubal wall. Salpingectomy has been described in literature as removal strategy. Recent literature describes removal of the devices starting from 10 weeks after placement up to 10 years following placement. [12] Lazorwitz et al. described earlier a combined hysteroscopy and laparoscopy technique. [9] All reports underline the need for gentle traction and the prevention of thermal damage when removing the device to prevent it from tearing. [6, 13] The only way to be sure the device will not tear is performing a hysterectomy or a salpingectomy combined with a large cornual resection. In a study from Sills et al., a questionnaire was developed for women who had undergone hysteroscopic sterilization using Essure^®^ followed by device removal surgery. A total of 3803 patients responded to the questionnaire and 2468 (64.9%) had undergone a hysterectomy. [14] Patients have chosen this surgery to prevent retained fragments of the device, despite the known higher cost, increased morbidity, prolonged recovery time, increased overall complication rate, and the risk of more blood loss during surgery associated with hysterectomy. However, also bilateral salpingectomy is a common approach for Essure^®^ removal. [8] If gynecologists understand the structure and function of the device, it is possible and feasible to perform nonhysterectomy removal. This reduces the risk of complications during and after surgery and the duration of the operation and recovery time.

## 4. Conclusion

This case report describes the tearing of both third markers of Essure^®^ ESS205 during removal surgery which resulted in a second removal surgery. It is important that gynecologists who perform Essure^®^ removal surgery are aware of the differences between the ESS205 and ESS305 model and are aware of which model is in situ before surgery. After removal, it is mandatory to identify both the third and fourth markers. Hysteroscopy and laparoscopy combined seems currently the best way to remove the devices. Hysterectomy is not indicated for complete removal, even after unsuccessful removal the first time. Using intraoperative fluoroscopy can prevent reoperation and hysterectomy. More research is needed to map out the incidence of retained fragments.
